# Barriers and drivers to stakeholder engagement in global mental health projects

**DOI:** 10.1186/s13033-021-00458-y

**Published:** 2021-04-03

**Authors:** Jill Murphy, Onaiza Qureshi, Tarik Endale, Georgina Miguel Esponda, Soumitra Pathare, Julian Eaton, Mary De Silva, Grace Ryan

**Affiliations:** 1 Department of Psychiatry, Faculty of Medicine, University of British Columbia, 2255 Westbrook Mall, Vancouver, BC V6T 2A1 Canada; 2 London School of Hygiene and Tropical Medicine, Keppel St, Bloomsbury, London, WC1E 7HT UK; 3 Department of Counseling and Clinical Psychology, Teachers College, Columbia University, New York, NY USA; 4 Institute of Psychiatry, Psychology and Neuroscience, King’s College London, 16 De Crespigny Park, London, SE5 8AF UK; 5 Centre for Mental Health Law and Policy, Indian Law Society, Pune, 411004 India; 6 Centre for Global Mental Health, Department of Population Health, London School of Hygiene and Tropical Medicine, Keppel St, Bloomsbury, London, WC1E 7HT UK; 7 Wellcome Trust, 215 Euston Road, London, NW1 2BE UK

**Keywords:** Global mental health, Stakeholder engagement, Implementation, Policy engagement, Low and middle income countries

## Abstract

**Background:**

Engagement with diverse stakeholders, including policy makers, care providers and service users and communities, is essential for successful implementation of global mental health interventions. Despite being a fundamental factor in the implementation process, evidence about challenges and drivers to stakeholder engagement is limited in the global mental health literature.

**Methods:**

We conducted semi-structured qualitative interviews with 29 recipients of Grand Challenges Canada Global Mental Health funding to assess barriers and drivers to global mental health implementation across a portfolio of projects. We used framework analysis to identify key themes related to implementation barriers and drivers. This paper reports on barriers and drivers to stakeholder engagement, with results related to capacity development and service delivery reported elsewhere in this journal.

**Results:**

Barriers and drivers to stakeholder engagement were identified across four themes: (1) Contextual Considerations, (2) Resources, (3) Participation, Uptake and Empowerment, and (4) Stigma. While complex contextual challenges create barriers, mechanisms such as formative research can facilitate a deeper contextual understanding that supports effective implementation planning. Limited financial and human resources and competing priorities can lead to substantial challenges. Investing in and leveraging existing local resources and expertise can help to mitigate these barriers. The challenge of achieving active participation from stakeholders and diverging expectations about the nature of participation were identified as barriers, while providing opportunities for meaningful participation and empowerment acted as drivers. Stigma at the institutional, community and individual level was also identified as a substantial barrier to engagement.

**Conclusion:**

The findings of this study are relevant to implementers in global mental health. They also have implications for global mental health funding agencies and policy organizations, who can support improved stakeholder engagement by investing in high-quality formative research, supporting capacity building for policy engagement, investing in longer-term funding schemes to support sustainable partnerships and scale-up, thus fostering successful engagement and supporting effective implementation of global mental health innovations.

## Background

Stakeholder engagement is essential for successful implementation in global mental health (GMH) [1] and has been identified as a priority by prominent GMH initiatives [2, 3]. Stakeholder engagement has also been identified as an essential skill for GMH implementers [4]. There is, however, little research that explicitly identifies barriers, drivers and best practices for stakeholder engagement in GMH.

‘Engagement’ in the GMH implementation context is defined by Roberts et al. [5] as “processes by which stakeholders are enabled to support or contribute to an intervention.” GMH implementation may involve a broad spectrum of stakeholders, including: policy makers (national and international), funders, service planners and managers, non-governmental organizations, service-users, their families and caregivers, members of the broader community, the media, service providers (specialist, non-specialist, and lay health providers), traditional healers, spiritual leaders, and representatives of other related sectors (e.g. education, housing, social services, etc.) [6–9].

Although literature detailing the processes of stakeholder engagement in GMH is not extensive, a number of barriers and drivers have been described. Engagement of policy makers is seen as fundamental to GMH implementation. Barriers to policy engagement include the low priority given to mental health at both national and international levels and the subsequent poor allocation of resources to mental health by national governments and international funding agencies [1, 8, 10, 11]. Additional factors such as competing priorities [9] and limited discretion for decision-making and priority setting [8] make policy engagement challenging. Despite these barriers, “policy windows” such as existing or emerging mental health policies and legislation may act as drivers for policy engagement [6].

Mental health care providers, working both within and outside of the health sector, are also an essential stakeholder group. Barriers to engaging primary care providers in the delivery of mental health interventions include reluctance to take on new tasks due to a large burden of work in busy primary care settings [9, 11], lack of adequate compensation, [6] and low perceived self-efficacy [12–14]. Activities designed to actively engage local providers, however, can act as important drivers, as observed by Davies and Lund who state that activities such as group and individual meetings and engaging with care providers prior to implementation are essential steps in promoting implementation success [6].

Despite extensive research from high-income countries on the engagement of service users, families and caregivers in mental health service implementation, research from LMICs in this area is limited [8, 15, 16]. In studies from India [17] and Ethiopia [18], barriers to mental health policy engagement by service users and caregivers included unfamiliarity by service users with the concept of and opportunities for policy engagement. Stigma, including in the health system, in the community and self-stigma was also identified as a barrier. Limited resources, including financial support, space and training and low access to mental health care also restricted the potential for engagement. Despite these considerable challenges, engagement with service users and their families and caregivers is essential for successful implementation, helping to reduce stigma, improve knowledge and attitudes towards mental health and increase help-seeking [9, 12]. Engaging service users and families in mental health policy and program development is also essential to improving mental health service access, availability and appropriateness [16]. Engagement with service users and community members, from the inception of research and implementation planning can promote an essential understanding of community concepts of mental health and the acceptability of planned interventions [12]. User-led research and participatory research methodologies such as community-based participatory research [19] and Theory of Change [20] can help to engage service users throughout the research process and ensure they are actively involved in priority setting, knowledge creation and dissemination.

Stakeholder engagement is an essential component of GMH implementation, and the gap in literature points to a need for research in this area. This study represents an opportunity to explore the perspectives of a group of GMH grantees working across different regions, target conditions and intervention models to further understand the challenges and drivers of stakeholder engagement in GMH.

## Methods

### Aims

This paper describes results of a qualitative analysis of barriers and drivers to stakeholder engagement in GMH as part of a multi-methods study examining barriers and drivers to implementation across a portfolio of GCC-funded projects [21–23]. Based on the results of a quantitative analysis of GCC-funded project outcomes using a portfolio-level Theory of Change framework [24], six key themes emerged as important to implementation success: (1) stakeholder engagement; (2) training providers; (3) supervision of providers; (4); detection of mental illness; (5) treatment of mental illness, and; (6) mental health promotion and awareness. These themes informed the design of this study, as described below. This paper is one of four reporting on this study, and describes results of the ‘stakeholder engagement’ theme.

### Data collection

**Fig. 1:**
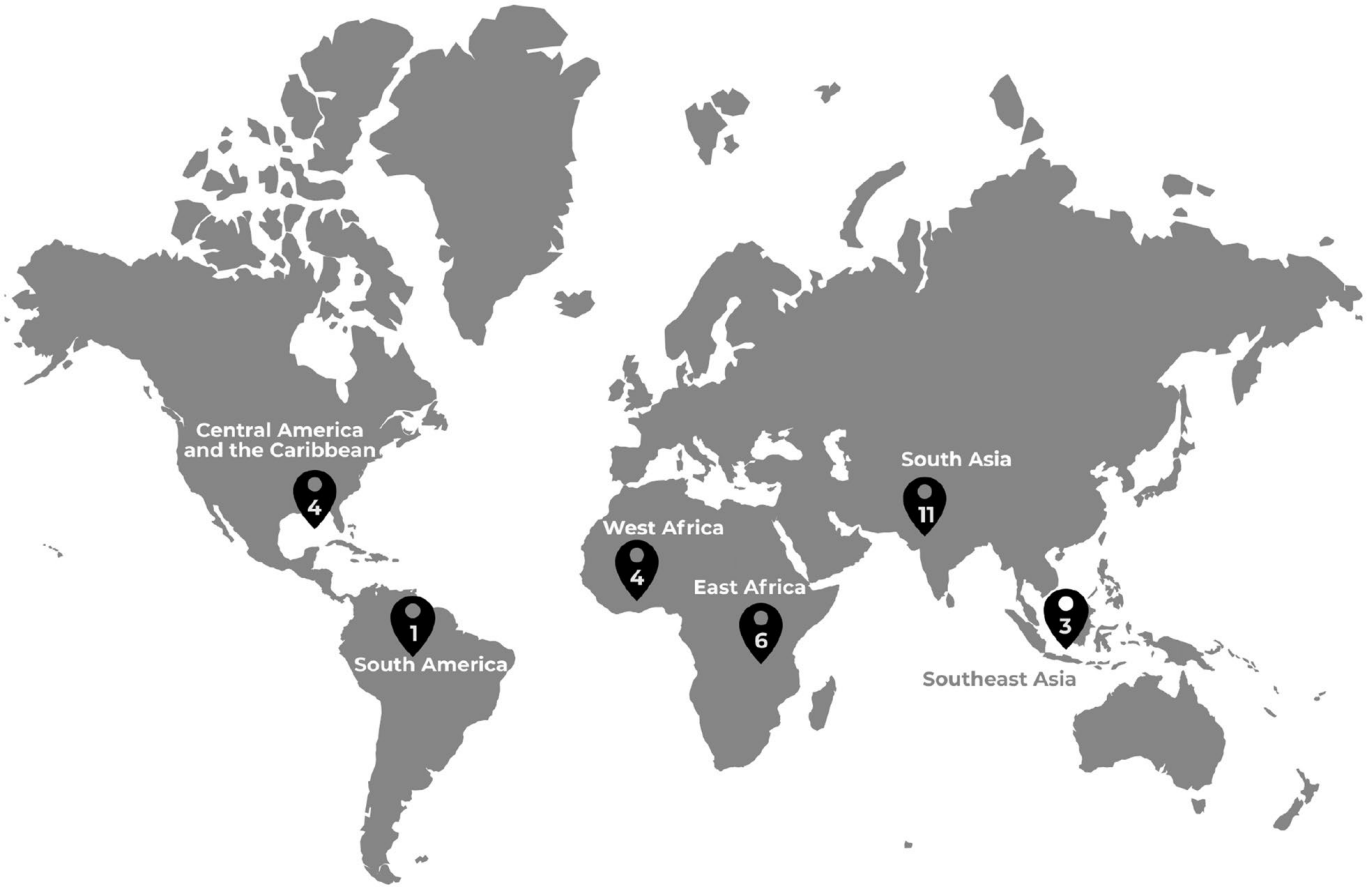
Geographic distribution of projects

This study took place between June 2014 and May 2017. Purposive sampling was used to recruit participants, with inclusion criteria comprising all current or former GCC Global Mental Health grantees who agreed to take part in the study. Sampling included GCC grantees, who were first approached during two Grand Challenges community meetings in the United States and United Kingdom for face-to-face interviews with members of the study team. Recruitment continued after the meeting with standardized participation templates, information sheets and consent forms sent by email. Interviews were conducted in-person or online using Skype. Of a total of 56 GCC Global Mental Health projects, 29 agreed to have their project lead or co-investigator participate. Participants were from GCC-funded projects in Latin America and the Caribbean (n = 4), South America (n = 1), West Africa (n = 4), East Africa (n = 6), South Asia (n = 11) and Southeast Asia (n = 3) (Fig. 1).

Study participants represented projects targeting a variety of mental health and substance use disorders [21], and were at different stages in their funding cycle with GCC at the time of interview. A semi-structured interview guide was developed, with questions corresponding to the six themes identified in the portfolio-level Theory of Change framework. Interviews allowed us to elicit barriers and drivers related to each theme and to explore each step on a collective Theory of Change (ToC) map representing projects in GCC’s Global Mental Health funding portfolio, as described elsewhere in this four-part series [21]*.* Drawing on the six implementation themes, grantees were asked to choose which implementation steps they felt were the most important to discuss in relation to their projects, and to describe what helped or hindered their success in completing this step. Members of the research team conducted the interviews, which were recorded with the consent of participants. Interviews ranged between 30 to 60 min. Ethics approval was granted by the London School of Hygiene and Tropical Medicine’s Research Ethics Committee (#7746 and #9945).

### Data analysis

We analysed transcribed interviews using framework analysis, which has been widely used in health policy research to identify barriers and drivers [25]. Three members of the research team (JM, OQ, TE) conducted coding using NVivo 11 software [26], with JM coding the full data set and OQ and TE each coding sixteen interviews. This approach was taken to balance time and resource constraints with methodological rigour via double coding for each transcript. Following immersion in the data JM developed an initial codebook, which was discussed and refined by the coding team. We coded three interviews using the refined codebook and conducted a coding comparison in NVivo 11. We then discussed areas of divergence, further refined the codebook, applied it to two additional interviews and developed a finalized version, which we applied to the remaining interviews. Because interview participants were asked to address more than one theme in their interviews, we coded across all six themes.

**Fig. 2:**
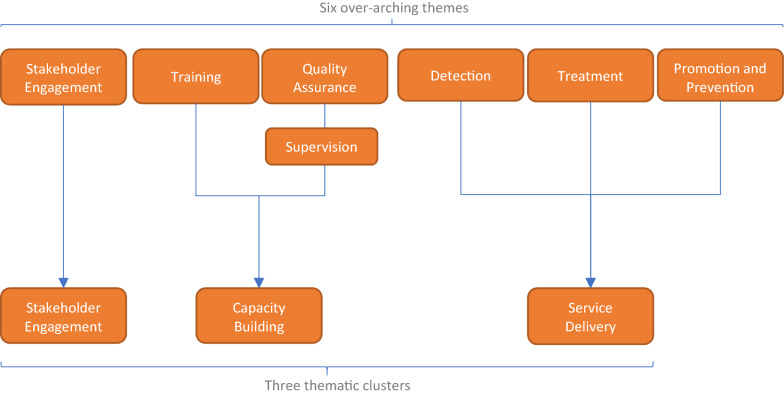
Thematic clusters

Based on previous research and emerging results during the analysis process, the research team agreed that the six key themes should be grouped into three thematic clusters: (1) Stakeholder Engagement; (2) Capacity building, and; (3) Service delivery (Fig. 2). This is consistent with previous research identifying common barriers and drivers to GMH implementation [5]. During analysis, preliminary findings further suggested that results were consistent with these three broad thematic categories.

Following the coding process, we used the codebook to create an analytic framework to identify emerging themes for each of the three thematic clusters. We populated the framework separately and then, using an iterative process, discussed and came to a consensus about predominant themes emerging from the codes. Each of three members of the study team (JM, OQ and TE) initially identified themes, and subsequently discussed and refined them until consensus was achieved. This paper presents findings for the stakeholder engagement cluster, with results from the other two clusters published as part of this series [22, 23].

## Results

### Stakeholder groups and the nature of engagement

Interview participants were either project leads or co-investigators leading global mental health projects in their respective settings. General characteristics of the projects that were implemented by the 29 interviewees are outlined in Table 1 below. Study participants described barriers and drivers to engagement with three broad categories of stakeholders: (1) service users and the community, (2) providers and, (3) policy makers. We define ‘service users and the community’ as those identified as recipients of care, their families or caregivers and the broader community in which they live and in which services are delivered. ‘Providers’ refers broadly to mental health service providers, ranging from specialist to non-specialist health workers, providers from other sectors (e.g. education, social services), and lay (e.g. traditional or peer) providers. ‘Policy makers’ refers to government officials at the national, regional or municipal level.

**Table 1:** General characteristics of global mental health projects being implemented by interview participant

Project characteristics	Qualitative study (n = 29) N (%)
Target disorder	
Common mental disorders	16 (55)
Behavioural and emotional disorders	13 (45)
Trauma and PTSD	7 (24)
Suicide and self-harm	7 (24)
Developmental disorders	7 (24)
Severe mental disorders	6 (21)
Epilepsy and seizures	5 (17)
Alcohol and substance use disorders	5 (17)
Dementia	2 (7)
All	3 (10)
Target population group	
Children and adolescents [1 month–14 years]	14 (48)
Young and old adults [15–60 years]	13 (45)
Vulnerable groups [e.g. conflict afflicted populations]	10 (34)
Women	9 (31)
Elderly [over 60 years]	6 (21)
Newborns [under 1 month]	2(7)
General population [all ages]	12 (41)
Region	
South Asia	11 (38)
Africa	10 (35)
Central America and the Caribbean	4 (14)
South East Asia	3 (10)
South America	1 (3)
Innovation components	
Capacity building	23 (79)
Detection, treatment, care and rehabilitation	22 (76)
Promotion and awareness	18 (62)
Stakeholder engagement	12 (41)

Study participants were asked to self-select the themes that they preferred to prioritize during their interviews. Participants were not given a pre-determined definition of stakeholder engagement, meaning that the parameters and processes described below emerged from the study participants’ own understanding and experience. The nature of engagement described by participants included advocacy with policy makers and efforts to encourage active participation and ownership of interventions by providers. Engagement by service users and the community was predominantly described by study participants in terms of uptake or participation in the study and/or intervention. Because participants were asked to describe barriers and drivers to engagement related to their GCC-funded projects, engagement in interventions also predominantly meant engagement in the research study through which the intervention was offered.

A number of cross-cutting themes emerged from the data, as described below. Results are summarized in Table 1.

### Contextual considerations—barriers

A number of contextual factors acted as considerable barriers to stakeholder engagement. Many of the GCC-funded projects take place in complex environments with fragile or emerging health systems, urgent and competing health priorities with limited funding and infrastructure, and unanticipated crises including political instability, conflict, natural disasters and epidemics. One participant describes the challenges of engaging stakeholders in this context:

*“It’s quite challenging because they’re picking up a broken or non-existent or collapsed health care system and trying to address huge problems, many of which are critical…I mean everything’s important but they are dealing with rebuilding everything. So it’s been really hard,”* (Participant 18)

Under-resourced health systems were identified as a challenge for initial and sustained engagement of service providers. Participants described the limitations of working with and training providers in the broader context of struggling health care delivery systems, challenging socioeconomic contexts, and barriers at the managerial and administrative level in the health sector. For example, the challenges of engaging with community health workers in the context of diminishing human and financial resources are described in the following quote:

*“…now you have a staff level of under 20 because some have resigned, some have gotten sick and some have retired and the economic environment that we’re in limits the replacement of human resources once they are lost in the health sector.”* [Participant 19]

Heavy workloads and competing priorities also posed challenges, with a majority of the studies using task-shifting models that involve primary care providers who are already working to capacity:

*“They [providers] were not really…interested or keen on getting an additional task added to their work list, because they’re generally seeing like 50–100 patients in a few hours’ time”* (Participant 20).

Fragile or emerging health systems, governance issues and limited government infrastructure act as a barrier to policy engagement. High turnover of bureaucrats and government officials make relationships, and thus buy-in, fleeting. Participants also describe the toll that limited infrastructure and funding can have on policy makers and their ability to engage. One participant states it is “like engaging with ghosts” (Participant 17).

Engagement was also challenging due to competing demands and priorities. Service users, for example, often have responsibilities that made it difficult for them to engage in interventions. Some were reluctant to engage without an incentive or compensation in cases where projects did not provide compensation for study participation. Others found that participating in the intervention took time away from essential activities such as income generation. This was particularly challenging in lower socioeconomic settings:

*“It was difficult for [service users] to come onboard for the sessions given the fact that they had to sell, to do other work before coming in. And because of the socioeconomic status and standards of our country, it was very difficult to tell someone stop what you are doing, where you get your income or where you get your daily bread and come for a session, a one hour or two hour session."* (Participant 11)

### Contextual considerations—drivers

Participants stressed the importance of a thorough understanding of the context of implementation and all key stakeholders involved, through formative research techniques such as situational and stakeholder analysis:

*“I think stakeholder analysis is very, very important…sometimes we collect prevalence only and health system challenges, but the structural arrangement of the community, the dynamics of the community sometimes we are not aware of. So the context- when we are talking about context, what do we really mean? Have we addressed the context or only part of the context?”* (Participant 22)

Participants also offered strategies to mitigate the barriers to engaging service providers when demanding workloads posed a potential barrier. They noted the importance of being realistic about demands, providing adequate support and structure, and streamlining mental health interventions with the existing tasks of providers.

### Relationships—barriers

A lack of reciprocity or shared vision in partnerships posed a barrier. Participants describe repeated attempts to engage with potential partners with little success:

“*…we’ve tried to engage with no results so for me the ultimate impact was still nothing. It’s just difficult in many ways you know, we’re trying to be the good partner but it takes two to tango, so we get nothing back and so in that sense it’s frustrating”* (Participant 9)

Time also emerged as a challenge related to fostering relationships. Participants described the time-consuming nature of stakeholder engagement, which is often incompatible with the timelines of research funding cycles:

*“So…thinking of this two-year grant process, when I think an organization like [organization] goes into a new area, we should really give ourselves much more time for engagement”* (Participant 6)

Capacity was also described as a barrier to engagement, particularly with policy makers. Participants explained that policy engagement might not fall within the usual scope or skillset of researchers, making the process burdensome:

*“I spent so much time, money and energy trying to lobby people, trying to meet people…trying to get people to meet with me. It was the most difficult aspect of the job, because it’s not something you are taught to do.”* (Participant 4)

Finally, trust is an additional barrier to relationship building and engagement. Communities might be reticent to participate in interventions due to harmful legacies of colonialism, racism and human rights abuses. When describing hesitancy to access health services among the population, one participant, for example, described mistrust of health workers by communities in the context of past negative experiences interacting with authorities and formal institutions:

*“So I think that is one thing missing in [this country]. Somehow we lack a trust between the parent and [the] professional*” (Participant 29).

### Relationships—drivers

Building on existing relationships and taking the time to foster long-term partnerships were described by participants as essential to achieving successful stakeholder engagement:

*“I think the key is relationships. You have to have relationships with the community with whom you’re going to be working with [sic]…Otherwise if you’re just going to come in, like fly in and fly out for a project, then I don’t think it’ll work very well …”* (Participant 1)

Having existing relationships at all levels of the health system and community is described as essential, helping programs to access stakeholders and begin the process of engagement:

*“We have a very good relationship with the district medical officer, the health district management team, and the community mobilisers, who are people who have been trained for a long time in the community…they know the community members, they know households, they can tell you the names of village leaders, so this really helpful because if you want to get to the community you know where to start, if you want to share the information they can tell you what road could be the right channel.”* (Participant 20)

Working with established collaborators means less time is required to build partnerships and establish trust. Engaging a project representative who is well-regarded in communities or by other local stakeholders can also help to build trust, confidence and ultimately promote engagement:

*“Having the right people in the country is the single, probably the most important thing. They have…this combination of seniority, competence, well known, well-loved and well trusted [sic], and this is- without that…you know, if you weren’t liked…we wouldn’t get it [buy-in]”* (Participant 9)

Leveraging existing resources and relationships within communities also promotes end user and community engagement:

“*Community engagement is actually doing just that, it’s getting the resources together that exist within the community but for our patients”* (Participant 19).

### Participation, uptake and empowerment—Barriers

Participants described the challenge of obtaining active participation from stakeholders, even when verbal agreements of collaboration had been obtained:

*“Like, you talk to the minister, you talk to everybody down from him to the…health centre, nurse- everybody say [sic] yes, definitely this is a good idea, but at the end of the day you end up doing it by yourself. People support the idea, but when it comes to actually implementing it, it’s just you”* (Participant 9)

Creating a sense of ownership and buy-in was also identified as a barrier to participation, particularly with regards to the prospect of long-term engagement and scale-up. This challenge is illustrated in the following quote about incentivizing provider participation:

*“We have to find out how we can make the deal sweeter for them, because eventually, if this has to be scaled up, they ‘have’ to take ownership of this and we have to find out how we can facilitate this process…So we have to kind of find out what will make them take this up from their level rather than someone telling them…”* (Participant 20)

Diverging expectations regarding the nature of mental health “treatment” was identified as a barrier to engaging with service users and the community. In many cases where psychosocial interventions were offered, service users and their families expected that their treatment would involve medications. Expectations related to treatment outcomes also acted as barrier. In some cases, with interventions for developmental disorders for example, parents entered the program with the expectation that their children would be “cured”. The expectation of curing instead of managing mental health conditions could lead to disappointment and threaten sustained engagement in interventions.

### Participation, uptake and empowerment—drivers

Creating opportunities for meaningful participation in programs helped to promote active engagement. For service users and the community, this can include their involvement in training and in program planning and delivery. One participant described how active participation of service users promoted their ongoing engagement and empowerment:

*“The service users… it’s been actually quite good, because…I think our project is the first time …where services users have …have been given so much prominence in the whole process and treated on par and equal, so this is for them a huge revelation and you know they are more than happy to engage because they… their voice is being heard.”* (Participant 1)

Making the work meaningful and empowering also acted as a facilitator to engaging service providers. In the case of community-based providers, their participation in programs might raise their status in their community. Formal health care providers might find participation meaningful when they develop new skills and see a difference in their patients. One participant described the enthusiasm of providers despite their heavy workloads:

*“What I saw among those nurses, they are enthusiastic about it, they were saying, like, you have opened our eyes for this. They were saying, like, we didn’t know that we could do this. They were asking questions, they were engaged, and all the… you go and read the WHO’s primary care challenge of integrating mental health into primary care and some other challenge, primary care workers are overworked, this is a extra work [sic], and so on, none of that was there!”* (Participant 9)

Participants described the importance of bottom-up approaches promoting policy maker buy-in. Disseminating evidence of program success and subsequent demand by providers and service users can help to encourage policy maker buy-in:

*“And so you put a policy document together, you send that to the [relevant ministry] that says this is a technique that works, we have the evidence, [providers] have heard about it. When the minister goes to meetings the [providers] jump on him and say listen, we’ve been doing this and it’s been working and you should listen. And he does!”* (Participant 8)

Similarly, the experiences of service users can promote engagement by others in the community. Participants described how service users who have had a positive experience tend to share their experience with peers, generating interest and promoting broader engagement in the intervention. One participant noted that:

*“The…successful cases have played an important role in promoting mental health inside the community”* (Participant 23).

### Stigma—barriers

Finally, stigma, including the low priority and low status of mental health both internationally and locally to stakeholder engagement acted as a barrier:

*“If you say I’m a psychiatrist, you say I’m a gynecologist, the respect is quite different…If you are to say I’m going to run a training of mental health and you are going to run a training on reproductive health, and you see the applicants, I’m sure you will have more applicants for reproductive health.”* (Participant 23)

Stigma and negative perceptions of people with mental health problems was also seen as a barrier to engagement with providers. Many providers consider working with people with mental illness to be particularly challenging or burdensome or were fearful of working with this population:

*“…we found out that this level of involvement of the nurse has been too low…because we found [it] pretty difficult for us to remove the huge burden of stigma in the nurses related to their fear, fear of the agitated patient, fear of violent patients. This is one of the reasons nurses have been pretty reluctant to involve themselves more in the process”* (Participant 23)

## Discussion

In the course of the broader study on barriers and drivers to GMH implementation [21], respondents self-selected themes and often spoke about more than one. Stakeholder engagement emerged as a prominent theme and was touched upon by a majority of respondents. Though our methodology did not involve quantifying the responses by theme, it was evident that responses related to stakeholder engagement made up a large proportion of data in the study, and that this was a priority among GMH innovators. The dearth of existing research in this area is somewhat surprising considering the prominence given to this theme in these interviews and the recognition of its importance by the broader GMH community [2, 3].

This study identifies numerous barriers and drivers related to stakeholder engagement in GMH implementation, suggesting that engagement is fundamental to implementation yet remains challenging to navigate. This highlights the strength of qualitative research methods in identifying and understanding factors that might otherwise go unnoticed or may be overlooked in other approaches to research. The following section details several broad themes and recommendations that may help to mitigate barriers and leverage drivers to promote successful GMH implementation. Cross-cutting themes are displayed in Table 2, with findings and recommendations displayed in Table 3.

**Table 2:** Barriers and drivers by cross-cutting theme

Cross-cutting themes	Barriers	Drivers
Contextual considerations	Complex environments Fragile, emerging or under-resourced health systems Competing priorities Low resources	Comprehensive contextual understanding Realistic demands, supportive structures and streamlining services
Relationships	Competition Lack of reciprocity Time Capacity Trust	Leveraging existing resources and relationships
Participation, uptake and empowerment	Achieving active engagement Diverging expectations	Meaningful participation Empowerment opportunities Bottom-up advocacy and communication
Stigma	Low status of mental health Negative perceptions of people living with mental illness

**Table 3:** Findings and recommendations

Findings	Recommendations
The importance of understanding context	Invest in high quality formative research
	Look for opportunities to “build back better”
The nature of engagement	Invest adequate time and funding to support engagement and trust-building
	Create opportunities for meaningful and active engagement by service users, providers and policy makers
	Promote formal and participatory engagement of service users and evaluate outcomes of this engagement
	Leverage existing resources and relationships
Communication and dissemination	Invest in informed mental health awareness raising and communication strategies
	Create mechanisms to support engagement from program inception
	Invest in capacity development opportunities to support knowledge translation and communications activities by researchers
	Invest in activities that promote mental health awareness and capacity building among policy makers

### The importance of understanding context

The study findings demonstrate the importance of developing a comprehensive contextual understanding prior to engaging in GMH implementation, which often takes place in challenging contexts with issues ranging from under-resourced health systems to natural disasters and conflict. Participants pointed to the importance of formative research, including situational and stakeholder analysis, to help identify factors in the broad implementation environment that might act as barriers and drivers. Situational analysis is recognized as an essential component of GMH implementation research [27].

Formative research can also help to navigate challenges related to the acceptability and appropriateness of interventions and to ensure that the adaptation of interventions responds to the needs of service users. For example, the challenge of service users being unable to participate in interventions when their participation meant they had to take time away from income generating activities demonstrates the clear importance of designing and adapting interventions so they are feasible for service users. Provider workloads also emerged as a barrier to engagement in this study and have been previously identified as a challenge in GMH [9, 11]. Consulting with providers prior to implementation about the acceptability of delivering mental health care is important [6, 12] and formative research can be used to identify barriers and drivers related to provider engagement.

Formative research with service users and the community is also essential to understanding factors influencing demand, help-seeking and access to care [28]. It is notable that engagement related to service users and the community as discussed by study participants predominantly focused on service uptake. This suggests that there is a need for more participatory, user-driven research in GMH, including the involvement of service users in priority setting and formative research. Though service user engagement has been emphasised as a key priority in GMH [2, 3], it has also been identified as an area that requires considerable strengthening [15]. There is an opportunity for enhanced engagement by investing in participatory and collaborative research approaches.

Formative research can also help to identify key stakeholders in the implementation environment and to assess barriers and drivers to engaging with them [29]. Makan et al. [9] describe the use of stakeholder analysis methodology to help identify stakeholders in the implementation context, their level of interest in mental health and opportunities for research uptake. This approach could help GMH implementers to better identify opportunities for engagement, and to better target their engagement activities to ensure maximum impact.

In addition to formative research, process evaluation may be used across the implementation trajectory to understand factors influencing the implementation of complex interventions [30]. Whereas situational analyses are helpful for capturing contextual factors that may influence implementation prior to undertaking research, process evaluations can be undertaken as part of feasibility studies or in congruence with randomized trials to assess barriers and drivers to implementation. Process evaluations can provide important insight into the interaction of context and intervention, helping to understand not only how context influences implementation, but also how the introduction of new interventions or programs may influence contextual factors and intervention outcomes [30].

Finally, although study participants emphasized the barriers posed by challenging contexts including war, insecurity and natural disasters, such crises may in fact lead to opportunities. Windows of opportunity for enhanced stakeholder engagement for GMH policy and program development may be generated by factors including the presence of international agencies and increased funding, the need to rebuild health and social support systems, and increased media attention thus creating the opportunity to ‘build back better’ [31, 32].

### The nature of engagement

The results of this study also demonstrate the importance of the nature of stakeholder engagement processes. The time required to engage with stakeholders, particularly policy makers, in the timeframe of research grant funding emerged as a barrier in this study. The challenge of building trust also emerged, further suggesting a need to invest time to foster trusting relationships based on common objectives. Initiating engagement activities early and taking time to develop a shared vision and sense of reciprocity among stakeholders is key [33].

These findings point to a need and opportunity in GMH funding. Making funding available to facilitate the development of partnerships and to conduct stakeholder engagement activities prior to the research stage could lead to research and implementation projects that are built on already-established collaborations. These types of funding opportunities could, for example, support integrated knowledge translation activities [34] that help to engage multiple stakeholder groups in the design and inception of GMH research and implementation programs, helping to build the foundation for ongoing collaboration.

The need to engage with all stakeholders in a way that promotes their active participation, collaboration and empowerment was a key theme and has previously been recognized as essential to GMH implementation [6, 9, 11, 35], with the active engagement of service users, for example, recommended to promote improved service acceptability and uptake [9]. Examples from this study demonstrate that providing opportunities for active participation in programs facilitated engagement and promoted empowerment among service users and providers, at times changing the status of stakeholders within their communities or organizations.

The benefits of active engagement with service users suggest that there is a need for enhanced formalized and participatory engagement processes in GMH. In a systematic review of service user and caregiver involvement in efforts to strengthen mental health systems in LMICs, Semrau et al. [16] found that there is a shortage of research in this area and identify the need for research that rigorously evaluates the impact of end user involvement on outcomes such as quality of life. There is a need for further efforts to formally engage service users in all aspects of research, project and program development, to identify best practices for meaningful participation and to evaluate the impact of this participation on service users and on project implementation.

Building on existing relationships was identified as an important facilitator of stakeholder engagement. Existing relationships with communities, organizations, and individuals at all levels of the health system were described as key to successful engagement. Involving stakeholders that are already known, trusted and well-regarded by communities was also described as a facilitator. Leveraging existing relationships can help to overcome the challenges of time and trust as described above, and can help to acknowledge existing capacity and knowledge within the context of implementation [33]. These existing relationships could also help to facilitate new collaborations and expanded partnership opportunities, with trusted collaborators helping to broker connections in new settings and with new partners [36].

### Communication and dissemination

The importance of communication and dissemination is also an important theme. Among service users and the community, diverging expectations about treatment processes and outcomes was identified as a barrier. Conversely, seeing or hearing about positive experiences with both programmes and treatment outcomes acted as a facilitator to engagement among all stakeholder groups. Engaging champions such as service users who have had positive experiences with an intervention or local leaders who lend credibility to a program may play a facilitating role in promoting uptake and successful implementation of mental health interventions [36]. These factors point to the importance of communication and awareness-raising about mental health and mental health services as a means of increasing engagement. Despite this fact, few of the programs included in this study included mental health promotion and awareness components [22]*.* This indicates a need for improved communication for mental health awareness-raising among stakeholders. Effective communications and awareness raising may also help to decrease stigma and improve service uptake [28].

More formal structures and processes can also be put in place to promote implementation, including by engaging stakeholders to act in a facilitating role throughout the implementation process. The integrated-Promoting Action on Research Implementation in Health Services (i-PARIHS) framework [37], for example, identifies facilitation as the central mechanism for promoting implementation, including via the engagement and ongoing support of external (e.g. research team members) and internal (e.g. representative of implementing organization) facilitators [38]. The engagement of formal implementation facilitators has been found to improve implementation outcomes [39], though may require additional resources to be sustained beyond the research funding period. Engaging both external and international facilitators can ensure that implementation processes are informed by a combination of technical expertise and rich contextual knowledge which, along with appropriate facilitation processes, may support implementation success.

Engaging with policy makers emerged as a challenge for study participants. Policy engagement requires specific skills and resources that may not be familiar or accessible to researchers. The potential for effective communication and dissemination of research results to encourage sustained engagement and buy-in from policy makers points to a need for mechanisms of support for researchers to engage in integrated knowledge translation activities by engaging key decision-makers from the inception of research [34]. Methods such as Theory of Change workshops [24] may facilitate engagement of policy makers and other stakeholders in program planning and priority setting from the outset [20]. This also suggests a need for researchers to seek out capacity building opportunities and to invest time to learn skills that enable them to effectively participate in policy engagement and communications activities. Similarly, while policy engagement is critical to GMH implementation, policy makers often have limited knowledge and understanding about mental health needs and challenges [8]. This indicates that there is also a need for improved capacity building activities that help to enhance knowledge and understanding of mental health among policy makers [40, 41]. GMH funding agencies have an important role in supporting policy engagement, and could help to promote policy engagement in GMH by facilitating and engaging in priority-setting exercises with policy makers and providing capacity building support to funded researchers. For example, GCC facilitated the use of Theory of Change methodology by integrating it as a funding requirement. Given the very low investment in mental health by donor agencies [42], increased investment by funding agencies would also help to increase prioritization of mental health by local governments, thus supporting more sustained engagement in mental health-related programs at the policy level (Table 3).

### Limitations

Through this study we sought to elicit perspectives of a diverse sample of GCC funded projects working in GMH implementation research. A potential limitation was our ability to only conduct interviews in the English language. This may have led to the omission of perspectives from non-English speaking grantees. Despite this, we did capture perspectives from a diversity of countries and regions (Fig. 1), including those that are non-Anglophone.

The objective of this study was to capture the perspectives of GCC-funded grantees. For this reason, we did not include perspectives of the stakeholders whose engagement is described as essential in this paper. Further research exploring implementation barriers and drivers from the perspectives of service users and the community, providers and policy makers would further enrich the understanding of factors influencing GMH implementation.

The institutional affiliation of many of the interviewers for this study may also have acted as a source of bias. A majority of interviews were conducted by staff and students at the Centre for Global Mental Health at the LSHTM, which, in collaboration with the World Health Organization, runs the Mental Health Innovation Network originally funded by GCC. To mitigate this risk, we explained to participants as part of informed consent that their confidentiality would be maintained and that no identifying features, including programme name or the country/ies in which their studies are based would be attributed to them in publications or other materials resulting from the study.

## Conclusions

This study has identified a number of barriers and drivers to stakeholder engagement for GMH implementation from the perspective of GCC-funded grantees and demonstrates the importance of stakeholder engagement at all stages of GMH research and implementation. The findings of this study have implications not only for GMH researchers and implementers, but also for funding agencies and organizations providing leadership in GMH.

The findings described above point to several opportunities for GMH funding and policy organizations to support stakeholder engagement for GMH implementation. Investing in and requiring high quality formative research as part of funded GMH research initiatives can help to promote a comprehensive contextual understanding prior to implementation. Recognizing the importance of early engagement, GMH funding agencies should support training and activities in integrated knowledge translation and participatory priority setting. The importance of leveraging existing relationships points to the need for long-term funding that supports multi-stage intervention, implementation and scale-up research and supports sustainable, long-term partnerships.

While stakeholder engagement for GMH is complex and often challenging, it is also fundamental to ethical GMH practice and to promoting improved mental health service access in LMICs. Engaging with stakeholders who will invest in, deliver and use mental health interventions is essential to ensure that these interventions respond to service user priorities and are acceptable to all stakeholders, promoting long-term adoption and sustainability. Understanding and thus anticipating barriers and drivers can help GMH implementers to be more successful in engagement activities. Opportunities also exist for GMH organizations, including funding agencies, to put in place structures and supports to promote the skills and conditions required for effective stakeholder engagement. Finally, though stakeholder engagement is essential to successful GMH implementation, research is limited in this area. Further research exploring factors influencing successful engagement, including from the perspectives of service users and communities, providers and policy makers, would make a significant contribution to the field of GMH implementation research.

## Data Availability

The qualitative data generated during the current study are not publicly available due to the sensitivity of discussions surrounding the performance of grantees’ projects but are available from the corresponding author upon reasonable request.

## Abbreviations

GCC: Grand Challenges Canada
GMH: Global Mental Health
LSHTM: London School of Hygiene and Tropical Medicine
